# Supplementary material for: Rifaximin Ameliorates Loperamide-Induced Constipation in Rats through the Regulation of Gut Microbiota and Serum Metabolites
Source: Nutrients. 2023 Oct 24;15(21):4502. doi: 10.3390/nu15214502 (PMC10648458; doi:10.3390/nu15214502)
Supplement: Supplementary file 1 [file nutrients-15-04502-s001.zip › Supplementary.pdf]

1. Supplementary methods S1

1.1 Sample preparation and extraction

1.1.1 Liquid samples class I

The sample stored at -80 °C refrigerator was thawed on ice and vortexed for 10 s. 50 µL of sample and 300 µL of extraction solution (CAN : Methanol = 1:4, V/V) containing internal standards were added into a 2 mL microcentrifugetube. The sample was vortexed for 3 min and then centrifuged at 12000 rpm for 10 min (4°C). 200 µL of the supernatant was collected and placed in -20 °C for 30 min, and then centrifuged at 12000 rpm for 3 min (4 °C). A 180 µL aliquots of supernatant were transferred for LC-MS analysis.

1.1.2 HPLC Conditions

All samples were acquired by the LC-MS system followed machine orders.The analytical conditions were as follows, UPLC: column, Waters ACQUITY UPLC BEH C18 1.8 µm□2.1 mm * 100 mm; column temperature, 40 °C; flow rate, 0.4 mL/min; injection volume, 2 µL; solvent system, water (0.1 % formic acid): acetonitrile (0.1 % formic acid); The column was eluted with 5 % mobile phase B (0.1 % formic acid in acetonitrile) at 0 minute followed by a linear gradient to 90 % mobile phase B (0.1 % formic acid in acetonitrile) over 11 minutes, held for 1 minute, and then come back to 5 % mobile phase B within 0.1 minute, held for 1.9 minutes, then rapidly return to starting conditions.

1.1.3 MS Conditions (AB)

The data acquisition was operated using the information-dependent acquisition (IDA) mode using Analyst TF 1.7.1 Software (Sciex, Concord, ON, Canada). The source parameters were set as follows: ion source gas 1 (GAS1),50 psi; ion source gas 2 (GAS2), 50 psi; curtain gas (CUR), 35 psi; temperature (TEM), 550 °C, or 450 °C; declustering potential (DP), 60 V, or−60 V in positive or negative modes, respectively; and ion spray voltagefloating (ISVF), 5000 V or−4000 V in positive or negative modes, respectively. The TOF MS scan parameters were set as follows: mass range, 50–1000 Da; accumulation time, 200 ms; and dynamic background subtract, on. The product ion scan parameters were set as follows: mass range, 25–1000 Da; accumulation time, 40

ms; collision energy, 30 or−30 V in positive or negative modes, respectively; collision energy spread, 15; resolution, UNIT; charge state, 1 to 1; intensity, 100 cps; exclude isotopes within 4 Da; mass tolerance, 50 mDa; maximum number of candidate ions to monitor per cycle, 12.

1.2 Analytical methods

The original data file acquisited by LC-MS was converted into mzML format by ProteoWizard software. Peak extraction, peak alignment and retention time correction were respectively performed by XCMS program. The “SVR” method was used to correct the peak area. The peaks with detetion rate lower than 50 % in each group of samples were discarded. After that, metabolic identification information was obtained by searching the laboratory’s self-built database, integrated public database, AI database and metDNA.

1. 2.1 PCA

Unsupervised PCA (principal component analysis) was performed by statistics function prcomp within R (www.r-project.org). The data was unit variance scaled before unsupervised PCA.

1. 2.2 Hierarchical Cluster Analysis and Pearson Correlation Coefficients

The HCA (hierarchical cluster analysis) results of samples and metabolites were presented as heatmaps with dendrograms, while pearson correlation coefficients (PCC) between samples were caculated by the cor function in R and presented as only heatmaps. Both HCA and PCC were carried out by R package ComplexHeatmap. For HCA, normalized signal intensities of metabolites (unit variance scaling) are visualized as a color spectrum.

1. 2.3 Differential metabolites selected

For two-group analysis, differential metabolites were determined by VIP (VIP > 1) and P-value (P-value < 0.05, Student’s t test). For multi-group analysis, differential metabolites were determined by VIP (VIP > 1) and P-value (P-value < 0.05, ANOVA). VIP values were extracted from OPLS-DA result, which also contain score plots and permutation plots, was generated using R package MetaboAnalystR. The data was log transform

(log2) and mean centering before OPLS-DA. In order to avoid overfitting, a permutation test (200 permutations) was performed.

1. 2.4 KEGG annotation and enrichment analysis

Identified metabolites were annotated using KEGG Compound database (http://www.kegg.jp/kegg/compound/), annotated metabolites were then mapped to KEGG Pathway database (http://www.kegg.jp/kegg/pathway.html). Significantly enriched pathways are identified with a hypergeometric test's P-value for a given list of metabolites.

2. **Table S1.** The altered microbial communities in genus levels between the LOP and RIF groups.

**Species Name**	**LOP Mean (%)**	**LOP SD (%)**	**RIF Mean (%)**	**RIF SD (%)**	***p* Value**
g__Akkermansia	1.488	1.575	10.960	9.146	0.030
g__Romboutsia	7.953	3.807	3.675	3.177	0.030
g__Dubosiella	8.098	6.152	0.027	0.046	0.002
g__Clostridium_sensu_stricto_1	4.896	2.215	0.002	0.002	0.002
g__Allobaculum	4.336	6.860	0.002	0.006	0.034
g__Bifidobacterium	4.267	2.059	0.016	0.019	0.002
g__norank_f__norank_o__Clostridia_UCG-014	3.832	2.150	0.003	0.003	0.002
g__Coriobacteriaceae_UCG-002	2.517	2.105	0.002	0.002	0.002
g__Faecalibaculum	2.090	1.297	0.363	0.698	0.015
g__Ruminococcus_gauvreauii_group	0.001	0.003	2.198	1.971	0.002
g__Blautia	0.016	0.018	1.672	2.613	0.005
g__Lachnoclostridium	0.032	0.021	0.632	0.877	0.007
g__Candidatus_Saccharimonas	0.498	0.349	0.083	0.105	0.015
g__unclassified_f__Ruminococcaceae	0.344	0.079	0.222	0.110	0.041
g__Parasutterella	0.485	0.723	0.022	0.023	0.007
g__Erysipelotrichaceae_UCG-003	0.000	0.001	0.369	0.392	0.001
g__Eubacterium_ruminantium_group	0.083	0.100	0.227	0.599	0.024
g__Alistipes	0.241	0.165	0.064	0.038	0.015
g__unclassified_c__Bacilli	0.197	0.066	0.034	0.036	0.002
g__Erysipelatoclostridium	0.003	0.003	0.218	0.211	0.007
g__unclassified_k__norank_d__Bacteria	0.134	0.104	0.042	0.038	0.041
g__Gordonibacter	0.012	0.013	0.153	0.121	0.006
g__norank_f__Erysipelatoclostridiaceae	0.000	0.000	0.106	0.172	0.004
g__Clostridium_innocuum_group	0.000	0.000	0.106	0.262	0.011
g__Eubacterium_nodatum_group	0.011	0.013	0.094	0.064	0.025

g__Lachnospira	0.092	0.082	0.000	0.000	0.004
g__Rothia	0.002	0.002	0.071	0.099	0.037
g__Eubacterium_brachy_group	0.050	0.051	0.000	0.000	0.001
g__norank_f__Christensenellaceae	0.043	0.023	0.005	0.008	0.005
g__Anaerofustis	0.000	0.001	0.040	0.044	0.002
g__Achromobacter	0.028	0.008	0.003	0.005	0.002
g__norank_f__norank_o__norank_c__Clostridia	0.029	0.009	0.000	0.000	0.001
g__Tuzzerella	0.018	0.026	0.008	0.022	0.040
g__Ruminiclostridium	0.022	0.016	0.003	0.006	0.006
g__unclassified_o__Coriobacteriales	0.024	0.023	0.000	0.000	0.004
g__unclassified_f__Erysipelotrichaceae	0.022	0.016	0.000	0.001	0.006
g__Globicatella	0.000	0.000	0.016	0.025	0.031
g__A2	0.001	0.002	0.014	0.018	0.047
g__Pygmaiobacter	0.009	0.005	0.002	0.003	0.009
g__Peptococcus	0.011	0.013	0.000	0.000	0.001
g__Rikenella	0.008	0.006	0.001	0.002	0.005
g__Eisenbergiella	0.009	0.010	0.000	0.000	0.004
g__Exiguobacterium	0.006	0.007	0.000	0.000	0.011
g__Plesiomonas	0.000	0.001	0.005	0.005	0.038
g__Pediococcus	0.005	0.005	0.000	0.000	0.011
g__norank_f__Sutterellaceae	0.004	0.002	0.000	0.000	0.003
g__Ideonella	0.003	0.004	0.000	0.000	0.030
g__Christensenella	0.002	0.002	0.000	0.000	0.028
g__Crenobacter	0.002	0.002	0.000	0.000	0.028
g__Bradyrhizobium	0.000	0.000	0.002	0.002	0.028

3. **Table S2.** The altered microbial communities in specials levels between the LOP and RIF groups.

**Species Name**	**LOP Mean (%)**	**LOP SD (%)**	**RIF Mean (%)**	**RIF SD (%)**	***p* Value**
s__Akkermansia_muciniphila	1.483	1.568	10.930	9.118	0.030
s__Romboutsia_ilealis	7.914	3.804	3.628	3.119	0.030
s__Lactobacillus_murinus	1.422	1.542	7.769	5.963	0.015
s__uncultured_bacterium_g__Dubosiella	8.093	6.145	0.027	0.046	0.002
s__uncultured_bacterium_g__Clostridium_sensu_stricto_1	4.862	2.215	0.001	0.003	0.002
s__Bifidobacterium_pseudolongum	4.238	2.041	0.012	0.016	0.002
s__unclassified_g__norank_f__norank_o__Clostridia_UCG-014	2.765	1.048	0.002	0.003	0.002
s__uncultured_bacterium_g__Coriobacteriaceae_UCG-002	2.517	2.105	0.002	0.002	0.002
s__Faecalibaculum_rodentium	2.081	1.288	0.363	0.698	0.015
s__uncultured_organism_g__Ruminococcus_gauvreauii_group	0.001	0.002	2.190	1.966	0.002
s__uncultured_bacterium_g__Blautia	0.008	0.016	0.927	1.109	0.013
s__uncultured_bacterium_g__norank_f__norank_o__Clostridia_UCG-014	0.925	1.162	0.000	0.001	0.001
s__gut_metagenome_g__Lactobacillus	0.158	0.149	0.607	0.533	0.021
s__unclassified_g__Blautia	0.007	0.004	0.745	1.562	0.002
s__unclassified_g__Lachnoclostridium	0.027	0.015	0.629	0.879	0.007
s__uncultured_bacterium_g__Candidatus_Saccharimonas	0.497	0.348	0.081	0.101	0.015
s__unclassified_f__Ruminococcaceae	0.344	0.079	0.222	0.110	0.041
s__uncultured_bacterium_g__Parasutterella	0.479	0.710	0.022	0.023	0.007
s__uncultured_bacterium_g__Erysipelotrichaceae_UCG-003	0.000	0.000	0.368	0.392	0.001
s__uncultured_bacterium_g__Eubacterium_ruminantium_group	0.083	0.100	0.227	0.599	0.024
s__unclassified_c__Bacilli	0.197	0.066	0.034	0.036	0.002

s__uncultured_bacterium_g__Lactobacillus	0.041	0.041	0.178	0.149	0.030
s__unclassified_g__Alistipes	0.161	0.139	0.037	0.025	0.025
s__unclassified_k__norank_d__Bacteria	0.134	0.104	0.042	0.038	0.041
s__uncultured_bacterium_g__Gordonibacter	0.012	0.013	0.153	0.121	0.006
s__uncultured_bacterium_g__Erysipelatoclostridium	0.000	0.000	0.131	0.166	0.011
s__uncultured_bacterium_g__Desulfovibrio	0.111	0.109	0.019	0.024	0.035
s__unclassified_g__Enterorhabdus	0.023	0.012	0.100	0.086	0.040
s__unclassified_g__Christensenellaceae_R-7_group	0.105	0.052	0.007	0.010	0.002
s__uncultured_bacterium_g__norank_f__Erysipelatoclostridiaceae	0.000	0.000	0.106	0.172	0.004
s__uncultured_bacterium_g__Eubacterium_nodatum_group	0.011	0.013	0.094	0.064	0.025
s__uncultured_bacterium_g__Clostridium_innocuum_group	0.000	0.000	0.104	0.258	0.011
s__uncultured_rumen_bacterium_g__norank_f__norank_o__Clostridia_UCG-014	0.095	0.119	0.000	0.000	0.001
s__unclassified_g__Lachnospira	0.092	0.082	0.000	0.000	0.004
s__unclassified_g__Rothia	0.002	0.002	0.071	0.099	0.037
s__uncultured_bacterium_g__Alistipes	0.056	0.047	0.003	0.005	0.002
s__uncultured_bacterium_g__norank_f__Erysipelotrichaceae	0.012	0.013	0.047	0.037	0.034
s__unclassified_g__Erysipelatoclostridium	0.002	0.002	0.050	0.072	0.006
s__uncultured_bacterium_g__Faecalitalea	0.000	0.000	0.051	0.110	0.031
s__uncultured_bacterium_g__Eubacterium_brachy_group	0.047	0.049	0.000	0.000	0.001
s__uncultured_Clostridiales_bacterium_g__norank_f__norank_o__Clostridia_UCG-014	0.046	0.047	0.000	0.001	0.006
s__uncultured_bacterium_g__norank_f__Christensenellaceae	0.040	0.022	0.005	0.008	0.005
s__Anaerofustis_stercorihominis_DSM_17244	0.000	0.001	0.040	0.044	0.002
s__Lachnospiraceae_bacterium_COE1	0.000	0.000	0.040	0.095	0.030
s__uncultured_Clostridiales_bacterium_g__Monoglobus	0.037	0.027	0.000	0.000	0.001
s__unclassified_g__Clostridium_sensu_stricto_1	0.032	0.018	0.000	0.001	0.001
s__unclassified_g__Achromobacter	0.028	0.008	0.003	0.005	0.002

s__Alistipes_sp._cv1	0.020	0.016	0.009	0.011	0.046
s__uncultured_bacterium_g__Tuzzerella	0.018	0.026	0.008	0.022	0.040
s__uncultured_organism_g__Ruminiclostridium	0.022	0.016	0.003	0.006	0.006
s__unclassified_o__Coriobacteriales	0.024	0.023	0.000	0.000	0.004
s__unclassified_f__Erysipelotrichaceae	0.022	0.016	0.000	0.001	0.006
s__unclassified_g__Bifidobacterium	0.020	0.020	0.000	0.000	0.001
s__uncultured_bacterium_g__norank_f__norank_o__norank_c__Clostridia	0.020	0.007	0.000	0.000	0.001
s__uncultured_organism_g__Defluviitaleaceae_UCG-011	0.001	0.004	0.019	0.021	0.009
s__uncultured_bacterium_g__norank_f__UCG-010	0.017	0.014	0.002	0.003	0.019
s__uncultured_Allobaculum_sp._g__Allobaculum	0.019	0.034	0.000	0.000	0.011
s__unclassified_g__Globicatella	0.000	0.000	0.016	0.025	0.031
s__uncultured_bacterium_g__Ruminococcus	0.001	0.002	0.014	0.027	0.046
s__uncultured_Desulfovibrionales_bacterium_g__norank_f__Desulfovibrionaceae	0.015	0.012	0.000	0.000	0.003
s__uncultured_bacterium_g__A2	0.001	0.002	0.014	0.018	0.047
s__uncultured_rumen_bacterium_g__Christensenellaceae_R-7_group	0.011	0.008	0.001	0.002	0.011
s__uncultured_bacterium_g__Pygmaiobacter	0.009	0.005	0.002	0.003	0.009
s__uncultured_bacterium_g__Peptococcus	0.011	0.013	0.000	0.000	0.001
s__uncultured_Clostridium_sp._g__norank_f__Eubacterium_coprostanoligenes_group	0.009	0.010	0.001	0.004	0.046
s__uncultured_prokaryote_g__Christensenellaceae_R-7_group	0.010	0.007	0.000	0.001	0.006
s__Clostridium_leptum_g__norank	0.008	0.009	0.001	0.002	0.048
s__uncultured_bacterium_g__Rikenella	0.008	0.006	0.000	0.001	0.002
s__uncultured_bacterium_g__Eisenbergiella	0.009	0.010	0.000	0.000	0.004
s__uncultured_organism_g__norank_f__norank_o__norank_c__Clostridia	0.009	0.006	0.000	0.000	0.001

s__Exiguobacterium_sp._AT1b	0.006	0.007	0.000	0.000	0.011
s__Pediococcus_pentosaceus_g__Pediococcus	0.005	0.005	0.000	0.000	0.011
s__Plesiomonas_shigelloides	0.000	0.001	0.005	0.005	0.038
s__uncultured_bacterium_g__Monoglobus	0.005	0.005	0.000	0.000	0.030
s__Christensenella_sp._Marseille-P2437	0.004	0.006	0.000	0.000	0.030
s__uncultured_Clostridium_sp._g__Christensenellaceae_R-7_group	0.004	0.005	0.000	0.000	0.030
s__unclassified_g__Eubacterium_brachy_group	0.003	0.003	0.000	0.000	0.030
s__uncultured_bacterium_g__Ideonella	0.003	0.004	0.000	0.000	0.030
s__Bacteroides_paurosaccharolyticus	0.000	0.000	0.003	0.003	0.030
s__Bradyrhizobium_elkanii_g__Bradyrhizobium	0.000	0.000	0.002	0.002	0.028
s__unclassified_g__Crenobacter	0.002	0.002	0.000	0.000	0.028
s__Christensenella_minuta	0.002	0.002	0.000	0.000	0.028

4. **Table S3.** The differential metabolites in the primary bile acid biosynthesis, bile secretion, steroid hormone biosynthesis and steroid biosynthesis metabolic pathways between the three groups.

Name	Quantitative analysis (CTR)		Quantitative analysis (LOP)		Quantitative analysis (RIF)		CTR vs LOP P-value	CTR vs LOP change in LOP	LOP vs RIF P-value	LOP vs RIF change in RIF
	Mean	SD	Mean	SD	Mean	SD				
7alpha,26-Dihydroxycholest-4-en-3-one	3808.500	4365.786	97483.333	41973.202	4435.000	3347.296	0.001	up	0.001	down
Glycochenodeoxycholic acid	264.667	117.437	4208.333	1333.798	482.083	514.599	0.003	up	0.003	down
Chenodeoxycholic acid	782.000	533.211	97038.333	62634.023	2994.500	2779.676	0.013	up	0.014	down
7alpha-Hydroxy-3-oxo-4-cholestenoic acid	2105.000	689.398	43878.333	21570.295	2515.000	2315.908	0.005	up	0.005	down
3,7-Dihydroxycoprostanic acid	227.147	230.767	8808.000	5665.365	305.533	201.131	0.014	up	0.014	down
Taurochenodeoxycholic acid	34216.667	29312.620	141716.667	77327.367	85050.000	29998.050	0.017	up	-	-
Taurocholic acid	13228.333	10924.267	14276.667	5588.090	34200.000	15746.492	-	-	0.025	up
Deoxycholic acid	1065.333	541.481	50633.333	36980.517	1424.000	649.855	0.022	up	0.022	down
Thyroxine	2978.333	366.574	1603.667	783.886	3090.000	445.062	0.006	down	0.004	up
leukotriene C4	2461.667	634.741	3601.667	817.323	2653.333	473.315	0.023	up	0.039	down

Lithocholic acid	30.400	18.505	1183.400	1078.320	24.657	25.800	0.047	up	0.046	down
L-Carnitine	6421.667	2348.509	3373.667	2285.975	3631.667	1076.465	0.046	down	-	-
Carnitine	61616.667	12332.626	39633.333	17472.912	55666.667	7452.427	0.033	down	-	-
Liothyronine	13793.333	4782.747	6919.833	4010.432	10285.000	5748.620	0.023	down	-	-
Tetracycline	2861.667	892.579	1371.883	972.934	1948.333	372.796	0.020	down	-	-
Vitamin D3	20.868	13.971	590.700	418.798	29.290	17.925	0.019	up	0.048	down
Calcitriol	38.250	12.170	764.817	549.941	80.400	73.147	0.017	up	0.017	down
Zymosterol	5.133	3.578	519.188	414.474	14.023	17.465	0.010	up	0.012	down
Androstanedione	6.383	7.481	377.167	184.969	17.028	22.069	0.021	up	0.022	down
Tetrahydrocortisol	7.335	5.168	446.017	309.044	7.327	5.140	0.023	up	0.028	down
Androstanolone	2855.833	1783.111	87.633	50.965	3411.500	2827.818	0.029	up	0.031	down
Testosterone glucuronide	11726.833	7244.799	1746.333	2289.250	8829.000	6478.687	0.004	up	0.005	down
5beta-Dihydrocorticosterone	192.292	6.000	1668.691	6.000	564.557	6.000	0.018	up	0.018	down
Estrone	37.793	6.000	2713.867	6.000	48.626	6.000	0.013	down	0.035	up
Estrone glucuronide	4.019	6.000	638.390	6.000	33.837	6.000	0.018	down	-	-
